# Genetic Diversity and Phylogeny of Antagonistic Bacteria against *Phytophthora nicotianae* Isolated from Tobacco Rhizosphere

**DOI:** 10.3390/ijms12053055

**Published:** 2011-05-12

**Authors:** Fengli Jin, Yanqin Ding, Wei Ding, M.S. Reddy, W.G. Dilantha Fernando, Binghai Du

**Affiliations:** 1 Shandong Key Laboratory of Agricultural Microbiology, College of Life Sciences, Shandong Agricultural University, Taian, Shandong 271018, China; E-Mails: jfl315@163.com (F.J.); dingyq6885@163.com (Y.D.); 2 Zunyi Tobacco Company, Guizhou 564700, China; E-Mail: dw6482@sina.com; 3 Department of Entomology and Plant Pathology, 209 Life Sciences Bldg, Auburn University, Auburn, AL 36849, USA; E-Mail: munagrs@auburn.edu; 4 Department of Plant Science, University of Manitoba, Winnipeg, MB R3T 2N2, Canada; E-Mail: dilanthafernando@yahoo.ca

**Keywords:** genetic diversity, antagonistic bacteria, *Phytophthora nicotianae*, tobacco, rhizosphere

## Abstract

The genetic diversity of antagonistic bacteria from the tobacco rhizosphere was examined by BOXAIR-PCR, 16S-RFLP, 16S rRNA sequence homology and phylogenetic analysis methods. These studies revealed that 4.01% of the 6652 tested had some inhibitory activity against *Phytophthora nicotianae*. BOXAIR-PCR analysis revealed 35 distinct amplimers aligning at a 91% similarity level, reflecting a high degree of genotypic diversity among the antagonistic bacteria. A total of 25 16S-RFLP patterns were identified representing over 33 species from 17 different genera. Our results also found a significant amount of bacterial diversity among the antagonistic bacteria compared to other published reports. For the first time; *Delftia tsuruhatensis*, *Stenotrophomonas maltophilia*, *Advenella incenata*, *Bacillus altitudinis*, *Kocuria palustris*, *Bacillus licheniformis*, *Agrobacterium tumefaciens* and *Myroides odoratimimus* are reported to display antagonistic activity towards *Phytophthora nicotianae*. Furthermore, the majority (75%) of the isolates assayed for antagonistic activity were Gram-positives compared to only 25% that were Gram-negative bacteria.

## 1. Introduction

Tobacco (*Nicotiana tabacum* L.) is an important economic crop in China. However, soil-borne fungal pathogens result in severe annual losses due to continuous cropping practices [1]. One such soil-borne pathogen, *Phytophthora parasitica* var. *nicotianae* is the causal agent of black shank disease of tobacco. This pathogen has a broad host range, although certain strains are specifically toxic towards tobacco [2]. Black shank is one of the most destructive and common tobacco diseases, and it can lead to losses at all growth stages ranging from minor injury to complete destruction of a tobacco plant [3]. Tobacco is highly susceptible to black shank, with common annual losses approaching 85% [4]. Once a field becomes infested with *Phytophthora nicotianae*, it cannot be eliminated; therefore the disease must be managed every year on a continual basis. For effective control of black shank, growers use a combination of crop rotation, cultivar resistance and fungicide application [5]. Nevertheless, black shank remains inadequately controlled and continues to be a major problem for farmers. Furthermore, increasing concerns over environmental problems (such as pesticide residues and water contamination are caused by chemical pesticide usage) and the emergence of the current chemicals resistant strains of *Phytophthora* supports the need for supplemental or alternative pathogen-control methods such as biocontrol [3].

Recently, antagonistic bacteria were used for biological control of soil-borne plant pathogens infecting plant roots [6–9]. An environmentally friendly approach to protecting plants from fungal pathogens is rhizobacterium-mediated biological control [10,11]. Numerous studies have demonstrated the ability of several antagonistic bacteria to suppress diseases caused by fungal plant pathogens [12–14]. Possible mechanisms antagonistic bacteria mediated disease suppression include direct antagonism of the pathogen by antagonistic bacteria [15–17], and the induction of systemic resistance in host plants which in turn, results in a reduction of disease development [18–21]. Antagonistic bacteria comprise a heterogeneous group of bacteria that can be found in the rhizosphere, at root surfaces and in association with roots. Within the last few decades, a large array of bacteria including species from the genera *Pseudomonas*, *Azospirillum*, *Azotobacter*, *Klebsiella*, *Enterobacter*, *Alcaligens*, *Arthobacter*, *Burkholderia*, *Bacillus* and *Serratia* have been reported to enhance plant growth. As biocontrol agents, isolates of *Pseudomonas fluorescens* and *Bacillus* have been the most studied and exploited [22–25]. Amongst their overall beneficial effects are the ability to deplete their immediate environment of available nutrients such as iron and to elute various metabolites possessing plant growth-antagonistic activity [26]. As well, pretreatment of seeds [6,27] and roots with antagonistic bacteria can induce the plant host defense reactions against fungal pathogens [28,29]. The study of antagonistic bacteria and their antagonistic potential is important to understand their ecological role in the rhizosphere and to elucidate their interaction with the host-plant.

To be considered as an effective antagonistic strain, bacteria must be rhizospherically competent, and capable of surviving and colonizing the rhizospheric soil [30]. Unfortunately, the relationship between antagonistic bacteria and plants can be unstable. Although promising results might be obtained *in vitro*, they are not necessarily reproducible under field conditions [31,32]. The variability with the performance of antagonistic bacteria may be attributable to various environmental factors that can determine antagonistic bacteria growth and as a result, affect their ability to exert a beneficial effect on the plant. Such environmental factors include climate, weather conditions (Temperature, Wind, Humidity, *etc*.), soil characteristics and the composition or activity of indigenous soil microbial flora. To achieve optimal growth conditions promoting interaction between antagonistic bacteria and nursery seedlings, it is important to determine how rhizobacteria exert their effects on plants and whether these effects are influenced by various environmental factors, including the presence of other microorganisms [33]. Therefore, it is necessary to develop efficient biocontrol strains under appropriate field conditions. One possible approach is to explore soil microbial diversity for antagonistic bacteria with plant growth promoting activities as such bacteria are assumed to be well adapted to the soil environment from which they are isolated [34].

A significant number of studies on tobacco rhizosphere microorganisms have focused on the isolation and identification of rhizosphere microbes by employing traditional physiological and biochemical methods [35,36]. Recently, molecular biology methods have been applied to investigations of the species diversity. PCR-based methods such as BOXAIR-PCR and 16S-RFLP (restricted fragment length polymorphisms analysis of 16S rRNA genes, also referred to as ARDRA: amplified ribosomal DNA restriction analyses) have proven suitable tools to examine microbial diversity in a wider range of environments, including soil and tobacco rhizosphere [37–42]. The objectives of this study are: (I) to screen stable and efficient antagonistic bacteria from the tobacco rhizosphere which can directly antagonize *Phytophthora nicotianae*; (II) to analyze the genetic diversity and phylogeny of culturable antagonistic bacteria from the tobacco rhizosphere; (III) to identify the species of the culturable antagonistic bacteria. To achieve these objectives, both molecular and standard cultivation techniques were used. Antagonistic bacteria were identified using a plate confrontation method. The BOXAIR-PCR and 16S-RFLP techniques were used to analyze the diversity of the culturable antagonistic bacteria. Finally, partial sequencing of 16S-rRNA genes was performed on culturable isolates to identify the species comprising the tobacco rhizosphere community.

## 2. Results and Discussion

### 2.1. Isolation of Bacteria

Bacteria were isolated from tobacco rhizosphere soil of different geographical areas. The CFU (Colony Forming Units) numbers of bacteria from all areas sampled were closely similar, at densities ranging from 6.3 to 7.8 log CFU/g (dry weight), and showed no significant regional differences. A total of 6652 isolates were obtained from the 55 different tobacco rhizosphere soil samples with each soil sample harboring a maximum of approximately 120 isolates.

### 2.2. Screening of Antagonistic Bacteria

**Figure 1.**
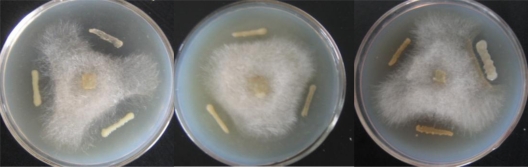
Screening of antagonistic bacteria used plate confrontation method in PDA (Potato Dextrose Agar) plates. In the middle of the agar plates are the Phytophthora nicotianae, around the Phytophthora nicotianae are parts of the antagonistic bacteria.

The bacterial detection and purification experiments were repeated three times and led to the eventual identification of a total of 267 bacteria strains demonstrating antagonistic activity against *Phytophthora nicotianae*. These isolates had varying zones of inhibition ranging from large to smaller inhibition zones (1–10 mm), with 31 bacteria strongly active, with inhibition zones larger than 10 mm. (Figure 1). Results showed that 4.01% of the culturable bacterial isolates were active against *Phytophthora nicotianae*, and 0.47% of the culturable bacterial isolates had a stable and efficient capability to control *Phytophthora nicotianae* and could be used for the prevention of Black shank disease during tobacco production.

### 2.3. BOXAIR-PCR Genomic Fingerprints

**Figure 2.**
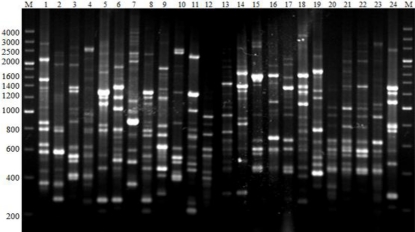
PCR DNA fingerprints generated with primer BOXAIR. Lanes (1–24) represent the BOXAIR groups 1, 2, 4, 5, 6, 8, 10, 11, 12, 13, 16, 18, 19, 20, 21, 23, 25, 26, 27, 29, 30, 31, 33 and 35. Lanes M are the marker 200 bp DNA ladder.

**Figure 3.**
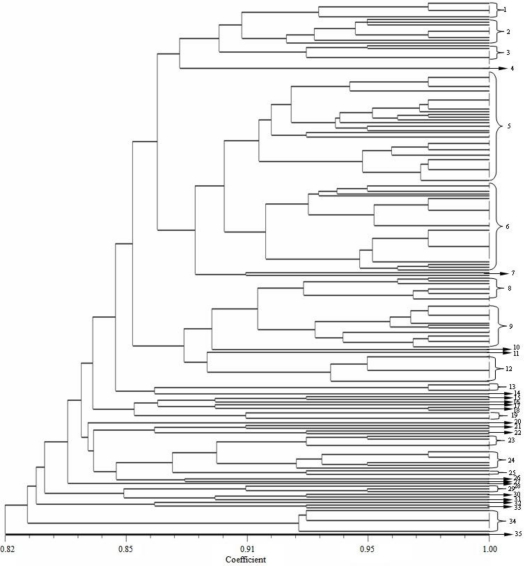
Dendrogram showing the relationship of 267 antagonistic bacteria based on BOXAIR-PCR fingerprints using cluster analysis.

BOXAIR-PCR performed with genomic DNA yielded fingerprints with 6 to 18 polymorphic bands ranging from 200 bp to 4000 bp (Figure 2). BOXAIR-PCR resulted in complex amplified banding patterns, reflecting a high degree of genotypic diversity among the antagonistic bacteria. These banding *patterns* were used to generate a dendrogram (Figure 3) that divided the 267 isolates into 35 clusters or genotypic groups at a level of 91% similarity and which clustered together at 82% similarity. This includes two large groups, group 5 and 6; 21 small groups and 12 groups which consisted of only a single isolate; groups 10, 11, 14, 16, 17, 20, 26, 27, 28, 31, 32 and 35. The BOXAIR-PCR patterns of the isolates belonging to groups 5 and 6 showed a more heterogeneous pattern than the other groups and contained 52 and 43 isolates respectively with both groups consisting of isolates from all nine geographical areas sampled. Overall, on the basis of the clustering results of BOXAIR-PCR, the antagonistic bacteria from different areas were separated into 35 different groups, with little correlation to agrotype or geographical area. The BOXAIR-PCR genomic fingerprints results indicate that the antagonistic bacteria may have adapted to their environment and display, abundant hereditary capacity in the long-term evolution process leading to a significant amount of genetic diversity.

### 2.4. 16S-RFLP Analysis

**Figure 4.**
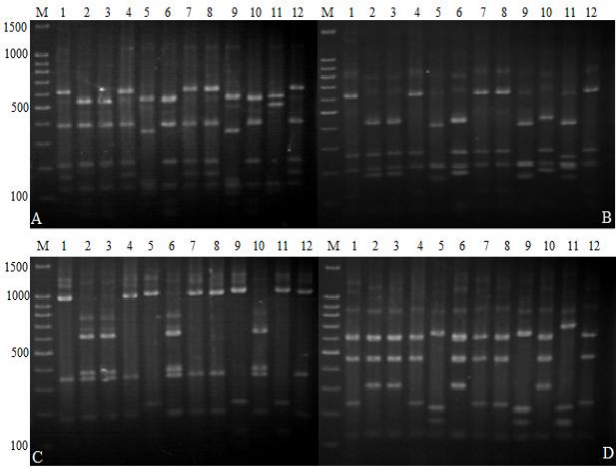
16S-RFLP patterns obtained from restriction digestion gel photos with Alu I, Hae III, Hinf I, and Msp I. Lanes (1–12) represent the 16S-RFLP groups 1, 3, 5, 6, 9, 11, 13, 15, 18, 21, 23 and 24. Lanes M are the marker 100 bp DNA ladder; A, B, C, and D represent restriction digestion gel photos with Msp I, Alu I, Hinf I, and Hae III.

The 267 antagonistic bacteria isolates were subjected to 16S-RFLP analysis by digestion of the amplified 16S rRNA gene with four restriction enzymes (*Alu* I, *Hae* III, *Hinf* I and *Msp* I). Similar banding patterns were obtained after combination analysis of the four independent digestions (Figure 4). These banding patterns were used to generate a dendrogram that separated the 267 isolates into 25 clusters or genotypic groups (Table 1) at the level of 88.5% similarity. As shown in Figure 4, each cluster displayed a specific banding pattern, and groups consisting of different species were clearly differentiated. Groups 1, 16, 20, 22, 24 and 25 each consisted of a single bacteria isolate; *Bacillus flexus*, *Serratia marcescens*, *Burkholderia arboris*, *Advenella incenata*, *Stenotrophomonas maltophilia* and *Myroides odoratimimus* respectively. On the other hand, groups 5 and 6 contained greater bacterial diversity with 57 and 46 antagonistic bacteria isolates, from the genera *Bacillus* and *Brevibacillus*, respectively. Therefore, these clustering results indicate that antagonistic bacteria are highly diverse at a phylogenetic level.

### 2.5. Sequencing and Phylogeny Analysis

**Figure 5.**
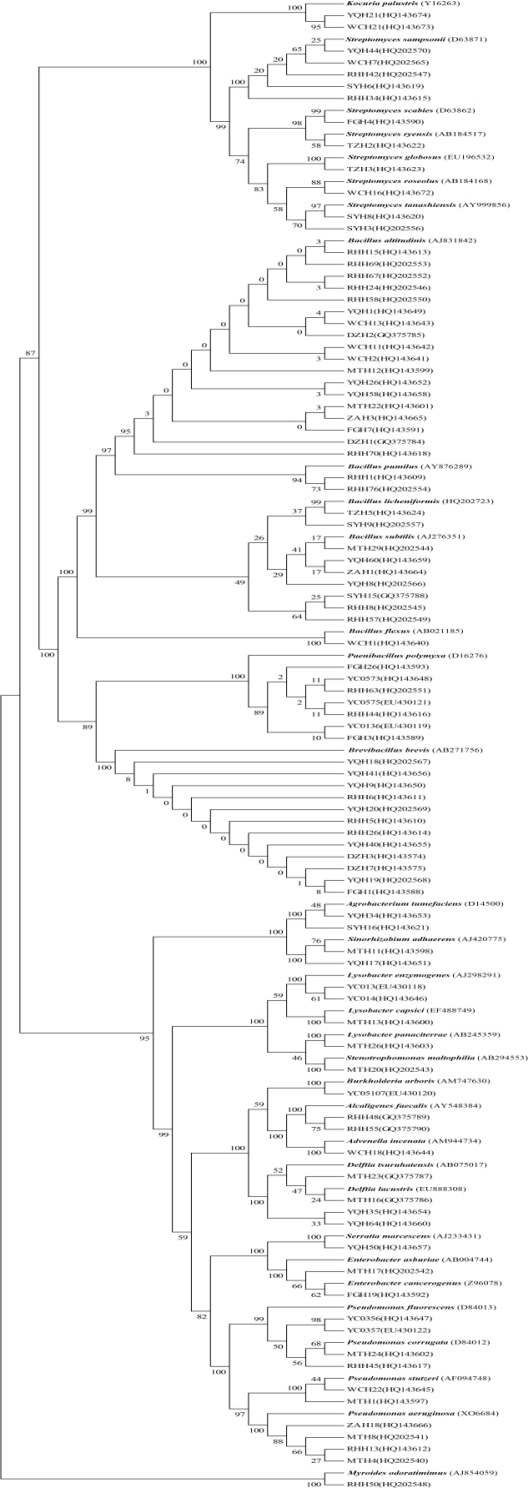
A neighbor-joining phylogenetic tree based on 16S rRNA gene sequences of antagonistic bacteria. The tree contains the closest type strain for each isolate. Bootstrap analyses were made with 1000 cycles.

According to the clustering results of BOXAIR-PCR and 16S-RFLP, representative isolates of each group were selected for partial 16S rRNA gene sequencing for comparison with annotated sequences in NCBI (Table 1). 16S rRNA partial sequences from 93 isolates were analyzed using the NCBI Blastn program. This indicated a high degree of similarity with a number of annotated sequences. For example; groups 13, 16, 17, 18 and 22, were identical to NCBI sequences, with 100% similarity (Table 1). A phylogenetic tree based on the 16S rRNA sequences of other antagonistic bacteria and our panel of 267 strains is shown in Figure 5. Ninety-three isolates clustered into 17 different genera which further divided into at least 33 different species. *Bacillus*, *Brevibacillus*, *Streptomyces* and *Pseudomonas* were the dominant genera identified, with 30, 12, 11 and 11 isolates, respectively. This includes, at least 5 *Bacillus*, 6 *Streptomyces* and 4 *Pseudomonas* species. In contrast, only a single *Brevibacillus* species, *Brevibacillus brevis* was identified.

### 2.6. Species Diversity of Antagonistic Bacteria

According to the clustering results of BOXAIR-PCR, 16S-RFLP and 16S rRNA sequencing, a wide variety of antagonistic bacteria were represented. The 267 isolates consisted of 17 different genera and at least 33 different species (Table 2). *Bacillus* species were dominant amongst the antagonistic bacteria with 93 total isolates and a minimum of 5 different species, particularly *Bacillus altitudinis*, with approximately 36 isolates identified to this species. This may be related to the high altitude of the sampling site. The other dominant genera identified were *Brevibacillus*, *Streptomyces* and *Pseudomonas* with 46, 46 and 32 isolates corresponding to a single, and a minimum of 6 and 5 species, respectively. Furthermore, a single species and strain isolated from *Serratia*, *Burkholderia*, *Advenella*, *Stenotrophomonas* and *Myroides* were present in the samples. These results indicate that there exists a great potential for species diversity among bacterial species exhibiting antagonistic activity against *Phytophthora nicotianae* in the tobacco rhizosphere. Otherwise, Gram-positive isolates were more frequently encountered, with 202 isolates, compared to 65 Gram-negative isolates.

## 3. Experimental Section

### 3.1. Rhizosphere Soil

Roots with adhering soil from tobacco plants were sampled into sterile bags for transport to the laboratory. 55 rhizosphere soil samples (yellow soil, clay, pH: 6.1∼6.5, lower soil fertility, lower organic matter: 8∼9 g/kg, total N: 0.6∼0.7 g/kg, total P: 0.6∼0.7 g/kg, total K: 34∼35 g/kg) from tobacco plants (YC-87) were collected from different areas of Zunyi city (105°36′–108°13′E, 27°8′–29°12′N; Elevation: 850∼1500 meters; Guizhou Province) between July 2008 and September 2008. Zunyi Region is an important tobacco production base in China, where soil-borne bacteria and fungal pathogen diseases may lead to severe annual losses in tobacco crops and revenues.

### 3.2. Isolation of Bacteria

Ten grams of each sample were dissolved in 90 mL of sterile water. To extract the rhizosphere microorganisms from the soil, samples were homogenized in a blender for 20 min. The samples were then serially diluted with sterile water and plated onto NA agar medium, PDA agar medium, Gause I agar medium, respectively. This procedure was repeated three times for each sample. Plates were incubated for two to five days at 30 °C. Bacterial isolates were obtained from agar plates containing 10 to 100 colonies.

### 3.3. Screening of Antagonistic Bacteria

A modified plate confrontation method was used to screen the resulting culturable bacteria for antagonistic activity of *Phytophthora nicotianae.* The *Phytophthora nicotianae* strain was isolated and screened from a Zunyi tobacco field in Shandong Province by our lab. PDA plates were inoculated with *Phytophthora nicotianae* hyphae in the middle of the plates. The plates were incubated at 28 °C for 1 to 2 days until the diameter of fungal hyphae reached approximately 2 cm. The plates were then inoculated with the bacterial strains 2 cm away from the pathogen using sterile toothpicks. Plates were incubated at 30 °C for 24 to 72 h. We determined the antagonism effect according to the zone of inhibition which was measured according to the method of Berg [43]. The detection experiment was repeated three times for each isolate after purification. Stock cultures were prepared in aseptic water supplemented with glycerol and kept at −80 °C prior to use.

### 3.4. Total DNA Extraction and 16S rDNA PCR Amplification

Genomic DNA of bacterial isolates was prepared according to the procedures of Murray and Thompson [44] with the exception that for Gram-negative bacteria, lysozyme was not used. The 16S rRNA gene was amplified from each isolate by PCR using the universal primers 27F (5′-AGAGTTTGATCCTGGTCAGAACGCT-3′) and 1492R (5′-TACGGCTACCTTGTTACGACTTC ACCCC-3′), which are designed to amplify universally conserved regions of the 16S rRNA gene and resulted in the amplification of an approximately 1500 bp PCR product. PCR was carried out in a Biometra TGRADIENT thermocycler (Whatman Biometra, G.ttingen, Germany). 25 μL reaction volume containing about 25 ng of genomic DNA, 2.5 U of *rTaq* DNA polymerase (TaKaRa Biotechnology (Dalian) Co., Ltd. China), 1× buffer (10 mM Tris-HCl [pH 8.3], 1.5 mM MgCl_2_, 500 mM KCl), 0.2 mM of each deoxynucleoside triphosphate (dNTP), and 0.4 μM of each primer. The PCR conditions were as follows: initial denaturation at 95 °C for 4 min followed by 32 cycles of denaturation at 94 °C for 45 s, annealing at 56 °C for 1 min and extension at 72 °C for 1.5 min followed by a final extension at 72 °C for 10 min. The presence and yield of specific PCR products (16S rRNA gene) was monitored by 1% agarose (w/v) gel electrophoresis at 120 V for 30 min in 1× Tris-Boric Acid-EDTA (TBE) buffer and stained with ethidium bromide (EB) for UV transillumination. Images of the agarose gels were taken using a UVP GelDoc-It Imaging System (UVP Inc. USA) under an ultraviolet lamp.

### 3.5. BOXAIR-PCR Genomic Fingerprints

BOX-PCR was performed as described by Rademaker and De Bruijn [45] of each isolate using the BOXAIR primer (5′-CTACGGCAAGGCGACGCTGACG-3′). PCR reactions were performed in a Biometra TGRADIENT thermocycler. 25 μL reaction volume contained about 50 ng of genomic DNA, 2.5 U of rTaq DNA polymerase, 1× buffer (10 mM Tris-HCl [pH 8.3], 1.5 mM MgCl_2_, 500 mM KCl), 0.2 mM of each dNTP, 0.4 μM primer, 2.5 μL DMSO, and 0.2 μL BSA (10 mg/mL). Conditions for BOXAIR-PCR were initial denaturation at 95 °C for 5 min, followed by 35 cycles of denaturation at 94 °C for 50 s, annealing at 53 °C for 1 min and extension at 65 °C for 8 min, and a final extension at 72 °C for 16 min. A 10 μL aliquot of the amplified PCR product was separated by gel electrophoresis on a 2% (w/v) agarose gels in 1× TBE buffer for 2 h at 100 V under refrigeration. Gels were stained with EB, and photographed under UV transillumination. The reproducibility of the results was verified by three independent experiments. Visible bands greater than 200 bp were used to construct the dendrogram. For each band, a binary data matrix was constructed on the basis of the presence or absence of each band (coded as 1 or 0, respectively). The patterns were used to construct a dendrogram using the unweighted pair group method with arithmetic averages (UPGMA), clustering algorithm using the NJ Coefficient along with the fine optimization option with NTSYS version 2.1 [46].

### 3.6. 16S-RFLP Analysis

PCR products were purified using the 3S Spin PCR Product Purification Kit (Shenergy Biocolor Bioscience and Technology Company, P. R. China) following the manufacturer’s instructions. Purified PCR products were digested with four enzymes; *Alu* I, *Hae* III, *Hinf* I and *Msp* I (TaKaRa Biotechnology (Dalian) Co., Ltd.) in separate digestion reactions. The selection of the four restriction enzymes was based on the studies of Laguerre *et al*. [47]. The digestions were performed for 4 h at 37 °C in 10 μL reaction volumes containing 5 μL of purified PCR products, 1 μL of the commercially supplied 10× buffers, 3.5 μL of water, and 0.5 μL (10 U/μL) of the restriction enzyme. Reaction products (10 μL) were run on a 2% (w/v) agarose gel in 1× TBE buffer for 2 h at 100 V under refrigeration. Agarose gels were stained, visualized and photographed as described above. The reproducibility of the results was verified in three independent experiments. Visible bands greater than 100 bp were used to construct the dendrogram. For each restriction enzyme’s band, a binary data matrix was constructed on the basis of the presence or absence of each band (coded as 1 or 0, respectively). The restriction digest patterns obtained with each enzyme were combined to obtain a single restriction digest pattern for each isolate. Computer-assisted evaluation of 16S-RFLP generated fingerprints was performed using the NTSYS 2.1 program as described above.

### 3.7. Sequencing and Phylogeny Analysis

According to the results of BOXAIR-PCR and 16S-RFLP cluster analysis, representative antagonistic bacteria were selected for sequencing reactions. Purified 16S rRNA PCR products obtained from the representative isolates were sequenced at BGI (Beijing). The nucleotide-nucleotide BLAST (blastn) of the NCBI (National Center for Biotechnology Information) database was used to identify highly similar 16S rRNA gene sequences. The 16S rRNA gene sequences were submitted to GenBank using SEQUIN. The accession numbers are HQ202540-HQ202570, HQ143574-143674 and EU430118-EU430122. The evolutionary distances were calculated using the software package TREECON version 1.3. The construction of neighbor-joining tree and bootstrap analysis of 1000 resamplings were performed using software MEGA 4 [48].

## 4. Conclusions

Antagonistic bacteria are important biocontrol agents of plant diseases. In this study, 4.01% isolates from the tobacco rhizosphere showed an antibiosis activity against *Phytophthora nicotianae*, and the antibiosis activity of 31 isolates was significant, with inhibition zones larger than 10 mm. Therefore, we conclude that antagonistic bacteria of *Phytophthora nicotianae* are common inhabitants of the tobacco rhizosphere. Furthermore, our sampling and detection approach proved to be an effective method of identifying potential biocontrol agents from the rhizosphere to control Black shank of tobacco.

The species diversity of culturable antagonistic bacteria from the tobacco rhizosphere was characterized in this study. Our results demonstrate that a total of 267 isolates were antagonists of *Phytophthora nicotianae* and that 35 BOXAIR-PCR clusters and 25 16S-RFLP groups were present among the 267 antagonistic bacteria isolates. BOXAIR-PCR showed that there was a low similarity level among the antagonistic bacteria isolates and that the genetic differentiation of individual strains was comparatively large. The 25 16S-RFLP groups represented bacteria from 17 different genera and at least 33 different species. This indicates that antagonistic bacteria possess a significant amount of genetic diversity and also have a strong adaptive response to their environment. Gram-positive isolates were more frequently encountered, with a total frequency of more than 75%. *Bacillus*, *Brevibacillus* and *Streptomyces* were the dominant genera present among antagonistic bacteria isolated from the tobacco rhizosphere, with a total frequency of 34.83%, 17.23% and 17.23%, respectively. For Gram-negative antagonistic bacteria, there were only 65 isolates found; from these, *Pseudomonas* was the dominant genera, with 11.99% total frequency.

The 16S rRNA genes of ninety-three representative antagonistic bacteria were sequenced, and the closest type strains of isolates were retrieved from NCBI. A phylogenetic tree based on 16S rRNA gene sequences of antagonistic bacteria and type strains were established. It was discovered that several isolates had am indeterminate classification according to the phylogenetic tree. Some isolates such as YQH35 and YQH64 were identified as *Delftia* isolates however, they showed closely related to *Delftia tsuruhatensis* with 97% similarity of the 16s rRNA gene, and therefore we suspect that they may represent new species of *Delftia*. Isolate RHH34 belongs to the *Streptomyces* genus and is similar to *Streptomyces sampsonii* with 97% similarity but with a high enough difference that it is also suspected to be a new species of this genus; Isolate RHH70 may be a new species of *Bacillus* related to *Bacillus altitudinis* (with 98% similarity); Isolates SYH15, RHH48 and RHH57 belong to the *Bacillus* genus and are similar to *Bacillus subtilis* with 98% similarity but are divergent enough to possibly be a new *Bacillus* species; Isolates YC0356 and YC0357 although closely related to *Pseudomonas fluorescens* with 96% similarity of the 16s rRNA gene, are also suspected to represent new *Pseudomonas* species. Detailed species characterization of these antagonistic bacteria based on morphological, physiological, biochemical and molecular biology tests are in process.

It was exciting to discover that 11.6% of the antagonistic bacteria were strongly active against *P. nicotianae*. To the best of our knowledge, this is the first time that *Bacillus altitudinis*, *Delftia tsuruhatensis*, *Delftia lacustris*, *Streptomyces roseolus*, *Burkholderia arboris*, *Lysobacter capsici*, *Sinorhizobium adhaerens*, *Stenotrophomonas maltophilia*, *Advenella incenata*, *Kocuria palustris*, *Agrobacterium tumefaciens* and *Myroides odoratimimus* are reported as antagonists of *Phytophthora nicotianae*. Specifically, this is the first study reporting antagonism of another microorganism by *Bacillus altitudinis*, *Advenella incenata* and *Myroides odoratimimus*. These antagonistic bacterial isolates demonstrate potential as future biocontrol agents.

Thirty-one isolates were selected for further characterization of their antagonistic and plant growth-promoting activity under greenhouse and field conditions (data not shown). In this study, all of the isolates were capable of antagonizing *Phytophthora nicotianae*. However, the success of biological approaches to control plant diseases and enhance plant growth is reliant upon their performance under field conditions. Raaijmakers and Berg [49,50] proposed that by matching rhizobacterium genotypes with crops for which they display a colonization preference, root colonization could be increased. The phenotypic and genotypic diversity which was found in natural populations and observed in our study of *Phytophthora nicotianae* antagonists offers a tremendous resource for the improvement of biological control strains. These prominent antagonistic bacteria are the leading candidates for further investigations into the biological control of this pathogen. Furthermore, we plan to test their effect on a wider scope of microorganisms which may broaden the antimicrobial spectrum especially the drug-resistant strains and applying the isolates to medical exploration. Detailed studies of the biochemistry of the antibacterial metabolites (antibiotics and extracellular hydrolytic enzymes) are in process, which we hope will be useful in our understanding of the mechanisms involved in biological control of diseases by antagonistic bacteria.

## Figures and Tables

**Table 1.** List of antagonistic bacteria obtained in this study representing each 16S-RFLP group and their closest affiliation according to sequencing of 16S rRNA gene.

**Group**	**No.**	**Isolate**		**Accession No.**	**Closest NCBI Strain and Accession No.**	**Similarity**
1	1	WCH1		HQ143640	*Bacillus flexus* GU339233	99%
2	12	RHH1		HQ143609	*Bacillus pumilus* FJ763645	99%
3	21	ZAH1		HQ143664	*Bacillus subtilis* GQ861470	99%
4	2	TZH5		HQ143624	*Bacillus licheniformis* GQ375247	99%
5	57		DZH2	GQ375785	*Bacillus altitudinis* HM582688	100%
			YQH26	HQ143652	*Bacillus* sp. HQ236080	99%
6	46		FGH1	HQ143588	*Brevibacillus brevis* AY591911	99%
7	13		FGH26	HQ143593	*Paenibacillus polymyxa* AM062684	99%
8	18		WCH7	HQ202565	*Streptomyces* sp. GU722183	99%
			TZH2	HQ143622	*Streptomyces ryensis* AB184517	99%
9	13		FGH4	HQ143590	*Streptomyces scabiei* HM018077	99%
			TZH3	HQ143623	*Streptomyces globosus* EU196532	99%
10	15		WCH16	HQ143672	*Streptomyces roseolus* AB184168	99%
			SYH8	HQ143620	*Streptomyces tanashiensis* FJ481625	99%
11	4		YQH21	HQ143674	*Kocuria palustris* EU379293	98%
12	17		RHH45	HQ143617	*Pseudomonas fluorescens* HM439651	99%
			MTH24	HQ143602	*Pseudomonas corrugata* AF348508	99%
13	8		ZAH18	HQ143666	*Pseudomonas aeruginosa* HJ472851	100%
14	7		WCH22	HQ143645	*Pseudomonas stutzeri* FJ959391	99%
15	4		FGH19	HQ143592	*Enterobacter cancerogenus* HQ154134	99%
16	1		YQH50	HQ143657	*Serratia marcescens* FJ360759	100%
17	7		MTH23	GQ375787	*Delftia tsuruhatensis* HM003215	100%
18	4		MTH11	HQ143598	*Sinorhizobium adhaerens* AJ420775	100%
19	3		SYH16	HQ143621	*Agrobacterium tumefaciens* FN433082	99%
20	1		YC05107	EU430120	*Burkholderia arboris* AB458219	99%
21	2		RHH55	GQ375790	*Alcaligenes faecalis* HM145896	99%
22	1		WCH18	HQ143644	*Advenella incenata* AM944735	100%
23	8		MTH13	HQ143600	*Lysobacter capsici* FN357198	99%
			MTH26	HQ143603	*Lysobacter panaciterrae* AB245359	99%
24	1		MTH20	HQ202543	*Stenotrophomonas maltophilia* FN645727	99%
25	1		RHH50	HQ202548	*Myroides odoratimimus* EU373431	99%
Total	267					

No.: The number of antagonistic bacteria; Isolate: The representative isolates of each group; Accession No.: GenBank accession number.

**Table 2.** List of the species richness of antagonistic bacteria obtained in this study.

**Genus**	**Species**	**Numbers of Strains**	**Percentage1**	**Percentage2**
*Bacillus*	*Bacillus altitudinis*	36	13.48%	34.83%
	*Bacillus* sp.	21	7.87%	
	*Bacillus subtilis*	21	7.87%	
	*Bacillus pumilus*	12	4.49%	
	*Bacillus flexus*	1	0.37%	
	*Bacillus licheniformis*	2	0.75%	
*Brevibacillus*	*Brevibacillus brevis*	46	17.23%	17.23%
*Paenibacillus*	*Paenibacillus polymyxa*	13	4.87%	4.87%
*Streptomyces*	*Streptomyces sampsonii*	11	4.12%	17.23%
	*Streptomyces roseolus*	3	1.12%	
	*Streptomyces globosus*	6	2.25%	
	*Streptomyces ryensis*	2	0.75%	
	*Streptomyces scabies*	5	1.87%	
	*Streptomyces tanashiensis*	6	2.25%	
	*Streptomyces* sp.	13	4.87%	
*Kocuria*	*Kocuria palustris*	4	1.50%	1.50%
*Pseudomonas*	*Pseudomonas aeruginosa*	9	3.37%	11.99%
	*Pseudomonas stutzeri*	7	2.62%	
	*Pseudomonas corrugate*	6	2.25%	
	*Pseudomonas fluorescens*	5	1.87%	
	*Pseudomonas* sp.	5	1.87%	
*Enterobacter*	*Enterobacter cancerogenus*	2	0.75%	1.50%
	*Enterobacter asburiae*	2	0.75%	
*Serratia*	*Serratia marcescens*	1	0.37%	0.37%
*Delftia*	*Delftia tsuruhatensis*	3	1.12%	2.62%
	*Delftia lacustris*	1	0.37%	
	*Delftia* sp.	3	1.12%	
*Burkholderia*	*Burkholderia arboris*	1	0.37%	0.37%
*Alcaligenes*	*Alcaligenes faecalis*	2	0.75%	0.75%
*Advenella*	*Advenella incenata*	1	0.37%	0.37%
*Lysobacter*	*Lysobacter enzymogenes*	3	1.12%	3.00%
	*Lysobacter capsici*	2	0.75%	
	*Lysobacter panaciterrae*	3	1.12%	
*Stenotrophomonas*	*Stenotrophomonas maltophilia*	1	0.37%	0.37%
*Sinorhizobium*	*Sinorhizobium adhaerens*	4	1.50%	1.50%
*Agrobacterium*	*Agrobacterium tumefaciens*	3	1.12%	1.12%
*Myroides*	*Myroides odoratimimus*	1	0.37%	0.37%
Total		267	100%	100%

Percentage1 denotes Numbers of antagonistic bacteria in the level of species/Total numbers of antagonistic bacteria; Percentage2 denotes Numbers of antagonistic bacteria on the genus level/Total numbers of antagonistic bacteria.
